# Zika Virus Persistently Infects and Is Basolaterally Released from Primary Human Brain Microvascular Endothelial Cells

**DOI:** 10.1128/mBio.00952-17

**Published:** 2017-07-11

**Authors:** Megan C. Mladinich, John Schwedes, Erich R. Mackow

**Affiliations:** a Department of Molecular Genetics and Microbiology, Stony Brook University, Stony Brook, New York, USA; b Genomics Core Facility, Stony Brook University, Stony Brook, New York, USA; c Molecular and Cell Biology Program, Stony Brook University, Stony Brook, New York, USA; Scripps Research Institute

**Keywords:** basolateral release, chemokine CCL5, IFN-β regulation, ISG15 induction, persistent infection, Zika virus, cell survival, human brain endothelial cells, innate immune regulation, transcriptome analysis

## Abstract

Zika virus (ZIKV) is a mosquito-borne *Flavivirus* that has emerged as the cause of encephalitis and fetal microencephaly in the Americas. ZIKV uniquely persists in human bodily fluids for up to 6 months, is sexually transmitted, and traverses the placenta and the blood-brain barrier (BBB) to damage neurons. Cells that support persistent ZIKV replication and mechanisms by which ZIKV establishes persistence remain enigmatic but central to ZIKV entry into protected neuronal compartments. The endothelial cell (EC) lining of capillaries normally constrains transplacental transmission and forms the BBB, which selectively restricts access of blood constituents to neurons. We found that ZIKV (strain PRVABC59) persistently infects and continuously replicates in primary human brain microvascular ECs (hBMECs), without cytopathology, for >9 days and following hBMEC passage. ZIKV did not permeabilize hBMECs but was released basolaterally from polarized hBMECs, suggesting a direct mechanism for ZIKV to cross the BBB. ZIKV-infected hBMECs were rapidly resistant to alpha interferon (IFN-α) and transiently induced, but failed to secrete, IFN-β and IFN-λ. Global transcriptome analysis determined that ZIKV constitutively induced IFN regulatory factor 7 (IRF7), IRF9, and IFN-stimulated genes (ISGs) 1 to 9 days postinfection, despite persistently replicating in hBMECs. ZIKV constitutively induced ISG15, HERC5, and USP18, which are linked to hepatitis C virus (HCV) persistence and IFN regulation, chemokine CCL5, which is associated with immunopathogenesis, as well as cell survival factors. Our results reveal that hBMECs act as a reservoir of persistent ZIKV replication, suggest routes for ZIKV to cross hBMECs into neuronal compartments, and define novel mechanisms of ZIKV persistence that can be targeted to restrict ZIKV spread.

## IMPORTANCE

ZIKV persists in patients, crossing placental and neuronal barriers, damaging neurons, and causing fetal microencephaly. We found that ZIKV persistently infects brain endothelial cells that normally protect neurons from viral exposure. hBMECs are not damaged by ZIKV infection and, analogous to persistent HCV infection, ZIKV constitutively induces and evades antiviral ISG and IFN responses to continuously replicate in hBMECs. As a result, hBMECs provide a protective niche for systemic ZIKV spread and a viral reservoir localized in the normally protective blood-brain barrier. Consistent with the spread of ZIKV into neuronal compartments, ZIKV was released basolaterally from hBMECs. Our findings define hBMEC responses that contribute to persistent ZIKV infection and potential targets for clearing ZIKV infections from hBMECs. These results further suggest roles for additional ZIKV-infected ECs to facilitate viral spread and persistence in the protected placental, retinal, and testicular compartments.

## INTRODUCTION

Zika virus (ZIKV) is the cause of encephalitis and fetal microcephaly outbreaks in Brazil and Puerto Rico (1–7). ZIKV is transmitted by mosquitoes, and ZIKV is predicted to cause >4 million clinical cases per year in the Americas (8). Unlike other mosquito-borne flaviviruses (FVs), ZIKV has unique properties that result in persistence (1 to 6 months) and sexual transmission (9, 10). ZIKV infects fetal and maternal cells, including neural stem cells, astrocytes, neuronal progenitor cells, microglia, Hofbauer cells, and endothelial cells (ECs) (6, 11–16). ZIKV crosses placental and blood-brain barrier (BBB) ECs, cells which normally prevent mixing of maternal and fetal blood and restrict access to adult and fetal neuronal compartments (17, 18).

Mosquito-borne FVs primarily cause acute febrile infections that are transient and resolve in ~2 weeks (19–21). Approximately 80% of FV infections are asymptomatic, with neurologic symptoms in <1% of Japanese encephalitis virus (JEV) and West Nile virus (WNV) cases and in an even smaller subset of dengue virus (DENV)-infected individuals. JEV causes more severe disease in children, and WNV encephalitis occurs primarily in elderly or inmunocompromised individuals (21, 22). DENVs have striking homology to ZIKV, cocirculate in humans and mosquitoes (20), but cause fever, hemorrhagic fever, and shock syndrome that are not central nervous system (CNS) associated and are instead linked to vascular leakage and EC dysfunction (19). Preexisting anti-FV immunity was recently found to enhance ZIKV pathogenesis in mice (23); however, among FVs, only ZIKV causes fetal demise and microcephaly during human infection (5, 16).

Hepatitis C virus (HCV) is a divergent blood-borne FV that persistently infects human hepatocytes and is transmitted from person to person (24, 25). Mechanisms of HCV persistence remain enigmatic but require balanced HCV replication in hepatocytes without cytopathology, as well as evasion of innate and adaptive immune responses that normally limit acute RNA virus infections (25–27). In humans, ZIKV is present in patient blood, tears, saliva, semen, and urine for <6 months (12). Cytotoxicity associated with ZIKV infection of neurons, neuronal progenitor cells, and trophoblasts (11, 14, 16) suggests that discrete cellular reservoirs enable acytopathic ZIKV replication and persistence. Although ZIKV does not establish HCV-like permanence, ZIKV persistence is unique to mosquito-borne FVs and likely to be a key to the ability of ZIKV to be sexually transmitted and enter neuronal compartments.

Innate and adaptive immune responses normally restrict acute FV replication and spread. Pretreating cells with type I interferon (IFN-α/β) blocks FV infection; however, FVs have developed mechanisms to restrict antiviral IFN-α/β, IFN regulatory factors (IRFs), and IFN-α receptor (INFAR) signaling defenses (20, 28–32). In murine settings, ZIKV fails to cause disease unless adapted or grown in immunocompromised (SJL), IRF^−/−^, or IFNAR-deficient (A129) mice (33–38). In these settings, ZIKV replicates to high titers, infects neurons, persists in tears, and causes optic damage, neurologic symptoms, placental damage, and fetal demise (12, 39). ZIKV infection of immunocompetent macaques results in persistent viremia (for <57 days in pregnant animals) with ZIKV present in cerebrospinal fluid, lymph nodes, saliva, and urine (40–42), similar to ZIKV persistence in humans.

ECs serve a primary barrier function that normally limits the access of blood constituents to the privileged neuronal, retinal, testicular, and placental compartments (18, 43–45). Brain capillaries form a BBB composed of unique brain microvascular ECs (BMECs), which restrict the emigration of immune cells and viruses to neuronal compartments (17, 46). Evidence that the BBB serves as a viral barrier is clear from findings that nonneurovirulent viruses injected into murine brains cause lethal neuronal pathology (21, 22). ZIKV infects patient ECs and human ECs derived from the aorta, brain, and lymphatic and umbilical vessels (6, 11, 12, 15, 39, 47, 48), suggesting that brain ECs may serve as potential conduits for ZIKV to bypass BBB restrictions.

Sites of ZIKV persistence and spread remain to be defined but appear central to the ability of ZIKV to breach barriers that normally restrict viral access to brain, testicular, and fetal tissues. We found that ZIKV (PRVABC59) persistently infects primary human BMECs (hBMECs) for >9 days postinfection (dpi) or following passage in hBMECs, without cytopathic effects. ZIKV was released from basolateral and apical surfaces of polarized hBMECs, but fail to alter hBMEC permeability. ZIKV infection of hBMECs was resistant to IFN-α added at >3 hpi, and IFN-β and IFN-λ were transiently induced but absent from cell supernatants for 1 to 9 dpi. Transcriptome analysis revealed that the chemokine CCL5 was highly induced and secreted by ZIKV-infected hBMECs and that infected hBMECs persistently induced and expressed cellular ISGs, including IFIT1 and MxA, as well as ISG inducers IRF7 and IRF9. ZIKV induced prosurvival factors and apoptosis regulatory genes ATF3, EGR1, IAP-2, and XAF1 and factors associated with HCV persistence, ISG15, HERC5, and USP18 (26, 27, 49). Our findings revealed that ZIKV persists in hBMECs by evading innate antiviral IFN and ISG responses and by uniquely balancing viral replication and cell survival in hBMECs. Persistently infected hBMECs provide a ZIKV reservoir that is uniquely situated to permit systemic ZIKV spread and basolateral emigration into neuronal compartments.

## RESULTS

### ZIKV (strain PRVABC59) productively infects and spreads in primary human brain ECs.

**FIG 1:**
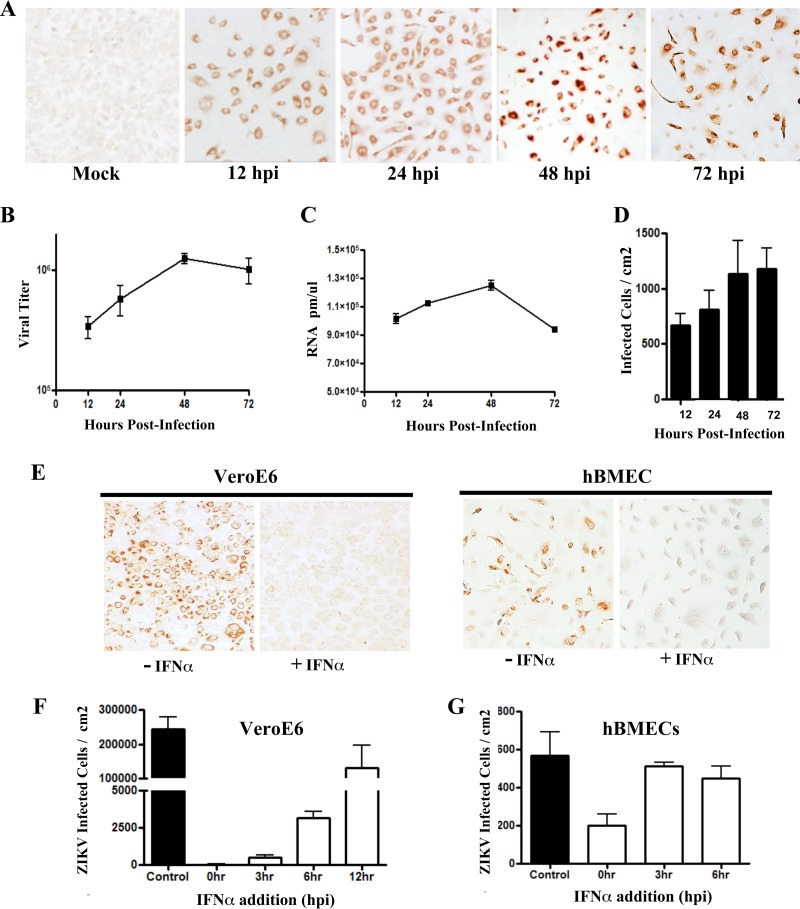
Zika virus infection of primary hBMECs. (A) Primary hBMECs were infected with ZIKV (PRVABC59) at an MOI of 10, and 12 to 72 hpi ZIKV antigen-positive cells were detected by anti-DENV4 HMAF. (B to D) Titers of ZIKV-infected hBMEC supernatants were determined in an FFU assay (B) and analyzed for cellular ZIKV RNA levels by qRT-PCR (C) and for infected cells (D). (E to G) hBMECs and Vero E6 cells were pretreated with IFN-α (1,000 U/ml) for 3 h prior to ZIKV infection (MOI, 10) (E), or IFN-α was added at the indicated time postinfection and infected Vero E6 (F) or hBMECs (G) were immunostained and quantitated 24 later.

ECs present in placental, fetal, and brain capillaries are infected in ZIKV patients (6, 12, 15), and ZIKV transmission to fetal neurons requires the virus to cross placental and brain EC barriers. hBMECs form a protective blood-brain barrier that restricts viral access to neuronal compartments (18), and mechanisms by which ZIKV bypasses the BBB remain to be defined. We initially assessed the ability of early-passage ZIKV (PRVABC59) to infect primary hBMECs (passages 3 to 10) by immunoperoxidase staining ZIKV-infected cells. We found that hBMECs were 80% infected by 12 to 24 hpi and remained infected for 1 to 3 dpi (Fig. 1A). ZIKV titers, RNA levels, and numbers of infected cells increased from 12 to 48 hpi (Fig. 1B to D), and inoculation of hBMECs with ZIKV at low multiplicities of infection (MOIs) of 0.1 to 1 resulted in viral spread within hBMEC monolayers for 1 to 3 dpi. ZIKV reached titers of 1 × 10^6^ focus-forming units (FFU)/ml in hBMECs (Fig. 1B), similar to ZIKV grown in IFN locus-deficient Vero E6 cells or a telomerase-immortalized human cerebral MEC (hCMEC/D3) cell line (see Fig. S1B and C in the supplemental material).

10.1128/mBio.00952-17.1 FIG S1  (A) Vero E6 cells were infected with ZIKV (PRVABC59) at an MOI of 10, treated with IFN-α (1,000 U/ml) at 3 to 12 hpi, and immunoperoxidase stained for ZIKV antigen at 24 hpi. (B) A telomerase-immortalized human cerebral microvascular EC cell line (hCMEC/D3) was infected with ZIKV (MOI, 5) and at 36 hpi, ZIKV antigen-positive cells were detected by immunoperoxidase staining. (C) hCMEC/D3 cells were infected with ZIKV (MOI, 5), and titers of infectious virus in supernatants were determined at 12 to 48 hpi. (D) hCMEC/D3 cells were pretreated with IFN-α (1,000 U/ml) for 3 h, or untreated, prior to ZIKV infection (MOI, 5). ZIKV antigen-positive cells were detected by immunoperoxidase staining at 24 hpi. Download FIG S1, TIF file, 30.7 MB. Copyright © 2017 Mladinich et al. 2017 Mladinich et al. This content is distributed under the terms of the Creative Commons Attribution 4.0 International license.

### ZIKV-infected hBMECs rapidly become type I IFN resistant.

The ability of ZIKV to replicate and spread in hBMECs, similar to infection of Vero E6 cells, suggested that ZIKV may potently regulate antiviral IFN responses. We added IFN-α exogenously at various times before or after ZIKV infection of hBMECs and Vero E6 cells (which are IFN-α responsive) and quantitated ZIKV-infected cells 24 hpi. Compared to untreated cells, prior addition of IFN-α inhibited ZIKV infection of both hBMECs and Vero E6 cells (Fig. 1E). Prior IFN-α addition also blocked ZIKV infection of human umbilical vein endothelial cells (HUVECs) and the hCMEC/D3 cell line (Fig. S1D). While simultaneous addition of IFN-α and ZIKV to Vero E6 cells blocked ZIKV infection, effects on ZIKV infection when IFN was added 3, 6, or 12 hpi were sequentially blunted (Fig. 1F; Fig. S1A). In contrast, hBMECs coadministered IFN-α and virus showed reduced ZIKV infection by ~70%, while IFN-α addition 3 to 6 hpi only reduced infected hBMECs by 10 to 20% (Fig. 1G). These findings indicate that ZIKV infection is inhibited by prior addition of IFN-α, but that by 3 hpi ZIKV-infected hBMECs are highly resistant to the effects of exogenously added type I IFN.

### ZIKV infection of hBMECs does not cause CPE.

**FIG 2:**
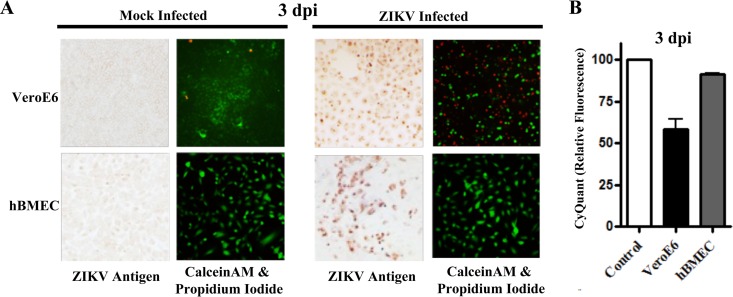
hBMECs are viable after ZIKV infection. (A) Vero E6 or hBMECs were infected with ZIKV (MOI, 10), and costained 3 dpi with calcein-AM (green [live cells])/propidum iodide (red [dead cells]). Following calcein-AM/PI staining, monolayers were fixed and immunostained for ZIKV antigen. (B) Viability of ZIKV-infected Vero E6 cells and hBMECs was assessed via CyQuant NF uptake 3 dpi, and results were compared to those for mock-infected controls.

ZIKV pathogenesis is linked to its capacity to damage neurons, and the cytopathic effect (CPE) of ZIKV on Vero cells is well established. However, we observed little, if any, cytopathology in ZIKV-infected hBMECs 1 to 3 dpi (~80% cells were ZIKV infected) (Fig. 1). We compared Vero E6 and hBMEC cell survival following ZIKV infection in propidium iodide (PI)/calcein-AM and CyQuant viability assays. We found that ~50% of ZIKV-infected Vero E6 cells were PI positive (dead) 2 to 3 dpi, with a dramatic reduction in viable cell uptake of calcein-AM (Fig. 2A). In contrast, ZIKV-infected hBMECs were nearly all PI negative and viable based on calcein-AM uptake 2 to 3 dpi (Fig. 2A). Similar viability was observed following ZIKV infection of the hCMEC/D3 cell line (Fig. S2A). CyQuant analysis confirmed that ZIKV-infected Vero E6 cells and hBMECs were, respectively, 55% and >95% viable 2 to 3 dpi (Fig. 2B). These findings indicate that ZIKV fails to cause cytopathic effects in hBMECs.

10.1128/mBio.00952-17.2 FIG S2  (A) hCMEC/D3 cells were mock or ZIKV infected (MOI, 10) and at 3 dpi or 3 days after hCMEC/D3 passage, cells were stained for ZIKV antigen or costained with calcein-AM/propidium iodide. (B) HUVECs and hCMEC/D3 cells were infected with ZIKV (MOI, 10) and analyzed at 9 dpi via immunoperoxidase staining. (C) Titers from supernatants of ZIKV-infected HUVECs and hCMEC/D3 cells were determined 3 days following cellular passage. Download FIG S2, TIF file, 23.8 MB. Copyright © 2017 Mladinich et al. 2017 Mladinich et al. This content is distributed under the terms of the Creative Commons Attribution 4.0 International license.

### ZIKV persistently infects hBMECs.

**FIG 3:**
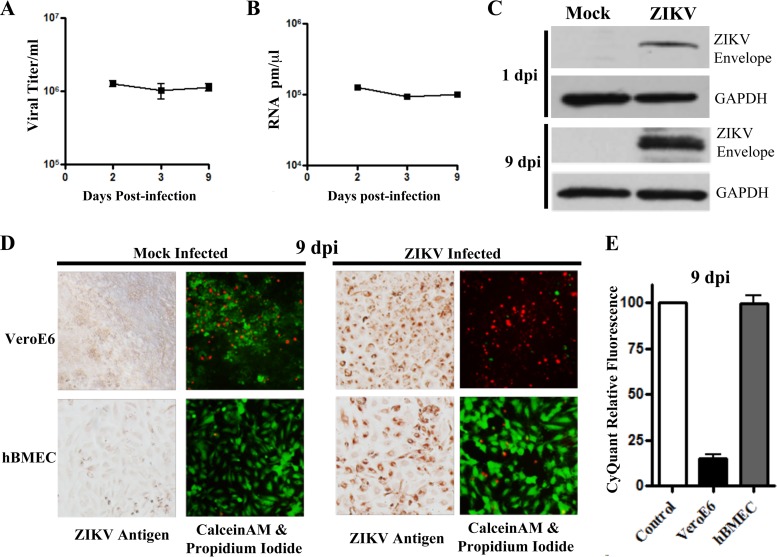
ZIKV persistently infects viable hBMECs 9 dpi. (A) hBMECs were infected with ZIKV as described for Fig. 2A, and titers present in cell supernatants were compared 2 to 9 dpi. (B and C) hBMECs were infected as described above, and cell lysates were analyzed for ZIKV RNA by qRT-PCR (B) and for ZIKV envelope protein (anti-Env) by Western blotting (C). Results were compared to those for the GAPDH controls 1 to 9 dpi. (D and E) Vero E6 cells or hBMECs were analyzed 9 dpi for ZIKV antigen and via calcein-AM/PI stain (D) or in CyQuant assays (E) for cell viability.

Given the survival of ZIKV-infected hBMECs and the continued spread of ZIKV within infected hBMECs, we analyzed the ability of ZIKV to persistently infect hBMECs. We replenished hBMEC culture medium every 3 days for infected or uninfected monolayers and evaluated viral titers and hBMEC viability 2 to 9 dpi. Consistent with ZIKV persistence in hBMECs, we observed ZIKV titers (~1 × 10^6^/ml) and RNA levels 9 dpi that were similar to those observed at 2 to 3 dpi (Fig. 3A and B), as well as expression of ZIKV envelope protein in hBMEC lysates (Fig. 3C). ZIKV-infected and mock-infected hBMECs were similarly PI negative, calcein-AM positive, and ~100% CyQuant viable 9 dpi (Fig. 3D and E). In contrast, ZIKV-infected Vero E6 cells (9 dpi) were nearly all PI positive, calcein-AM negative, and only ~10% viable by CyQuant analysis (Fig. 3D and E). ZIKV also persistently infected HUVECs and hCMEC/D3 cells 9 dpi based on immunoperoxidase staining, with no apparent CPE (Fig. S2B). These findings indicate that ZIKV persistently infects hBMECs without significantly diminishing hBMEC viability.

### ZIKV-infected hBMECs are viable following passage.

**FIG 4:**
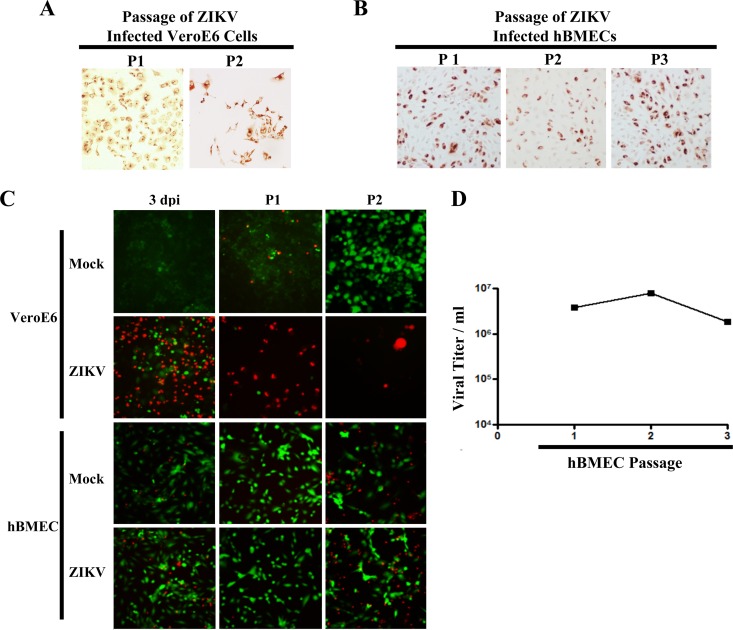
ZIKV-infected hBMECs are viable and productive following cellular passage. (A and B) ZIKV-infected Vero E6 cells or hBMECs (MOI, 10) were trypsinized and passaged (1:3) 3 dpi and every 3 days thereafter. ZIKV-infected passaged hBMECs were detected by immunostaining. (C) Cells were infected and passaged as for panels A and B, and cell viability was assessed via calcein-AM/PI staining and fluorescent image overlay. (D) ZIKV titers in supernatants of hBMECs consecutively passaged 1 to 3 times (every 3 days).

ZIKV antigen-positive Vero E6 cells adhere as monolayers regardless of their viability. However, trypsinizing ZIKV-infected Vero E6 cells resulted in <50% of cells being able to reattach to monolayers, and virtually all cells were PI positive after 3 days and unable to be passaged further (Fig. 4A and C). In contrast, trypsinization of ZIKV-infected hBMECs resulted in the propagation of viable ZIKV antigen-positive hBMECs through >3 passages (PI negative, calcein-AM positive) (Fig. 4B and C). Passage of highly infected hBMECs resulted in continued division and the production of ZIKV titers of ~2 to 5 × 10^6^/ml (Fig. 4D) with little or no cytopathology, similar to results in control hBMECs (Fig. 4C). Thus, ZIKV-infected hBMECs are viable following cellular passage and persistently produce ZIKV progeny.

### Global transcriptional changes in ZIKV-infected hBMECs.

Persistent ZIKV infection and spread suggests that ZIKV regulates antiviral responses and fosters hBMEC survival. We kinetically analyzed global transcriptional responses of hBMECs to synchronous ZIKV infection (80% infected at 1 dpi) (Fig. 1A). Human gene-level transcriptome arrays (Affymetrix, with >20,000 genes) were used to quantitate ZIKV-induced transcriptional changes in hBMECs compared to responses in mock-infected hBMECs. Selected, transcriptional changes are presented in Table 1, with complete data files available in the NCBI GEO database (GSE98889). ZIKV-infected hBMECs transiently induced IFN-β and IFN-λ at 1 to 2 dpi, and a subset of ISGs was highly induced 1 to 9 dpi (Table 1). Consistent with IFN-λ and ISG induction, IRF1 was transiently induced (1 to 2 dpi) while IRF7 and IRF9 were constitutively induced 1 to 9 dpi (50). ISG15, ISG15 family ligases (HERC5/6), and ISG15 proteases (USP18/41), which are implicated in HCV persistence (26, 27, 51), were also induced 1 to 9 dpi (Table 1). Transcription factors ATF3, EGR1, and TRAF1, which are linked to cell survival (52–54), were upregulated 39- to 301-fold (1 to 2 dpi), and ATF3 was induced 1 to 9 dpi. ZIKV also induced apoptosis-inhibitory and -regulating factors IAP2 and XAF1 (55) 5- to 201-fold (1 to 9 dpi) (Table 1). Two genes associated with cell permeability, Rnd1, a constitutively activated Rho GTPase (56), and ARHGAP26, a Rho-activating factor (57), were also induced in ZIKV-infected hBMECs. Chemokine CCL5/RANTES was highly induced (40- to 2,327-fold) 1 to 9 dpi, along with a subset of moderately induced CXCL10/11 and CCL20 chemokines (Table 1) (GEO GSE98889). Selected genes identified as upregulated by array were transcriptionally verified 1 to 9 dpi based on quantitative real-time PCR (qRT-PCR) analysis (Fig. S3). ZIKV-directed hBMEC transcriptional responses suggest that ZIKV evades antiviral IFN and ISG responses while engaging cell survival genes and pathways to establish persistence in hBMECs.

10.1128/mBio.00952-17.3 FIG S3  hBMECs were ZIKV infected as described for Fig. 1A. RNAs were purified from cell lysates at 1 to 9 dpi, and the induction of the cellular genes identified as induced by Affymetrix arrays (Table 1) (GEO GSE98889) were assayed by qRT-PCR and compared to RNA from mock-infected hBMECs harvested at the same time points. Download FIG S3, TIF file, 50.8 MB. Copyright © 2017 Mladinich et al. 2017 Mladinich et al. This content is distributed under the terms of the Creative Commons Attribution 4.0 International license.

**TABLE 1:** Global transcriptional responses of ZIKV-infected hBMECs

Category and protein	Fold induction versus controls at:					Confirmed by qRT-PCR
	12 hpi	1 dpi	2 dpi	3 dpi	9 dpi	
Interferons						
IFN-β		142		7	4	X
IFN-λ1		238	212	4		X
IFN-λ2		11	10			
IFN-λ3		23	23	2		
ISGs						
IFIT1	62	734	1,086	650	2,071	X
IFIT2	17	1126	869	68	58	X
IFIT3	6	226	434	130	282	
IFITM1		3	5	5	8	
MX1	36	515	1,081	575	209	X
OAS2	6	271	961	475	737	X
RSAD2^a^		57	193	106	616	
ISG15 and related						
ISG15		28	64	43	90	X
HERC5	5	451	292	52	46	X
HERC6		36	43	20	26	
USP18		3	4	7	13	X
USP41		3	6	7	13	
IRFs						
IRF1		114	24			
IRF7		5	9	6	10	
IRF9		6	7	3	3	
Transcription factors						
ATF3	4	301	101	7	7	
EGR1	7	168	155	20		
TRAF1		86	39	3		
JUNB	4	14	15			
Apoptosis regulatory factors						
BIRC3 IAP2		35	43	5	6	X
XAF1	5	62	201	86	50	X
Permeability						
Rnd1	3	169	80		5	X
ARHGAP26 RhoAct		12	8		2	
Chemokines and related						
CCL5/RANTES	9	2,327	1,914	352	40	X
CXCL10		247	179	35	60	X
CXCL11		305	244	57	7	
CCL20		385	126	8		
IL-1		20	11	5	3	
IL-6		10	11	3	2	

^a^ MX2 and OAS1 were similarly induced.

### Analysis of hBMEC responses to ZIKV infection.

**FIG 5:**
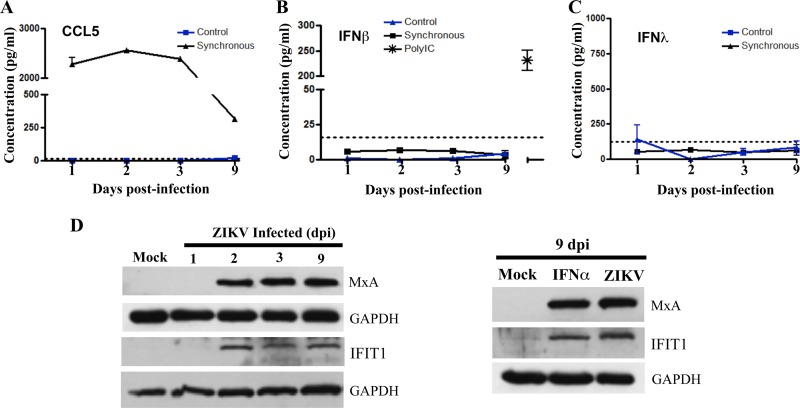
Analysis of cellular protein expression in ZIKV-infected hBMECs. (A to C) hBMECs were mock infected or infected with ZIKV, and 1 to 9 dpi supernatants were analyzed in an ELISA (R&D Systems) for CCL/RANTES (A), IFN-β (B), and IFN-λ (C) levels relative to antigen standards. As an hBMEC IFN-β response control, we transfected hBMECs with poly(I/C) (1 µg/ml) and Fugene6 at 3:1 and evaluated secreted IFN-β levels in supernatants via ELISA (36 h posttransfection) (D) Western blot analysis of MXA and IFIT1 genes, and GAPDH controls, in lysates from mock-infected or ZIKV-infected hBMECs (1 to 9 dpi). IFIT and MxA protein levels in ZIKV-infected hBMECs 9 dpi versus results 6 h post-IFN-α treatment (1,000 U/ml).

Analysis via an enzyme-linked immunosorbent assay (ELISA) determined that CCL5 was highly secreted into the supernatants of ZIKV-infected hBMECs at all time points from 1 to 9 dpi (Fig. 5A). Despite high levels of IFN-β secreted by hBMECs in response to poly(I/C) (Fig. 5B), we failed to detect IFN-β or IFN-λ secretion from ZIKV-infected hBMECs 1 to 9 dpi (Fig. 5B and C). In contrast, induced ISGs MxA and IFIT1 were expressed in ZIKV-infected hBMECs 2 to 9 dpi (Fig. 5D) and at levels similar to ISG expression levels induced by IFN-α treatment of hBMECs (Fig. 5E). Distinct from CCL5 secretion, these findings suggest that ZIKV uniquely regulates IFN-β/λ expression or secretion posttranscriptionally and further demonstrate that ZIKV evades antiviral functions of highly induced and expressed ISGs to persist in hBMECs.

### hBMEC monolayers are not permeabilized by ZIKV infection.

**FIG 6:**
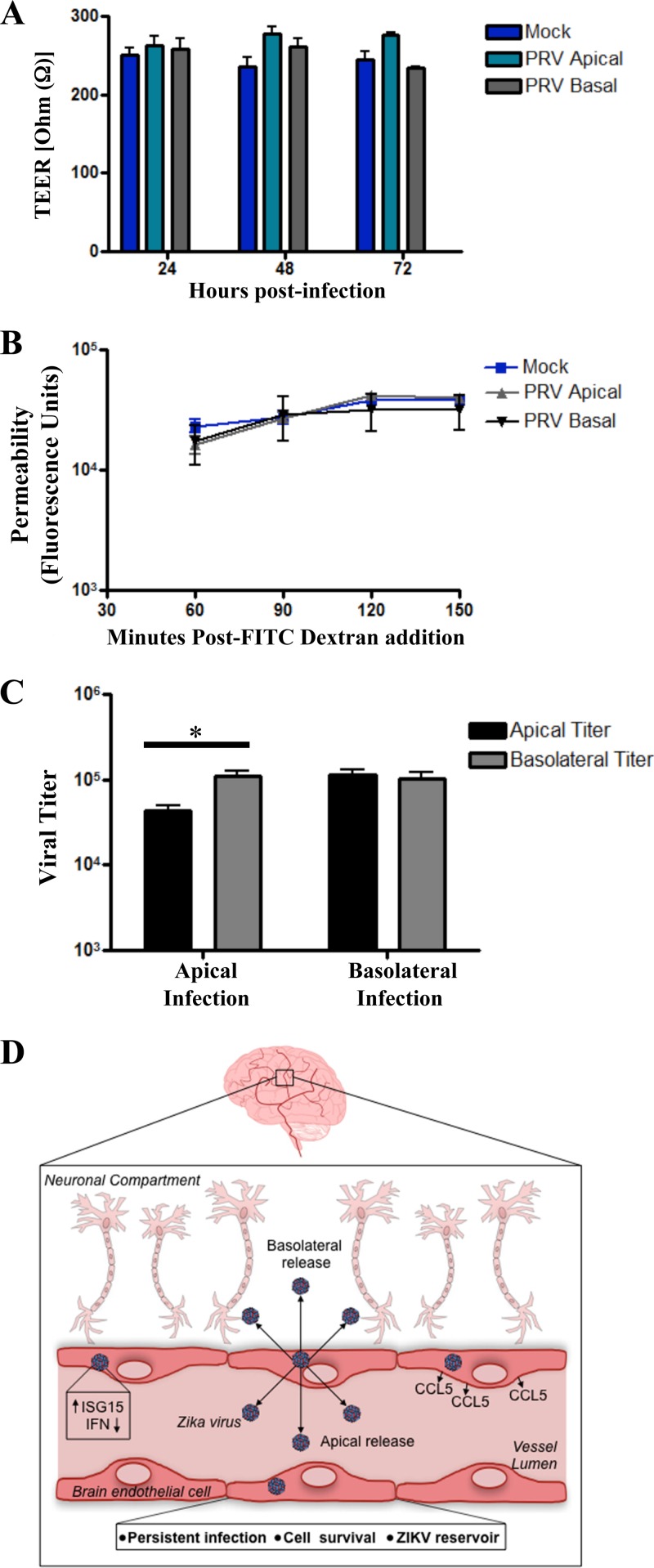
ZIKV-infected hBMECs release ZIKV basolaterally. (A) Polarized hBMECs, grown for 5 days in Transwell plates, were apically or basolaterally infected with ZIKV (MOI, 5) in triplicate, and TEER was measured 1 to 3 dpi. To demonstrate monolayer barrier function, EDTA was added (10 mM for 10 min) to hBMEC monolayers; this resulted in an ~100-Ω reduction in TEER. (B) hBMECs apically or basolaterally infected with ZIKV were assayed for permeability to FITC-dextran (40 kDa), which was added to apical medium at 3 dpi; fluorescence over time was measured in the lower chambers. (C) hBMECs grown on Transwell inserts for 5 days were evaluated for TEER. Cells were apically or basolaterally infected (MOI, 5) with ZIKV, and titers present in apical and basolateral supernatants were quantitated at 1 dpi. (D) Potential model of the spread of ZIKV systemically and to neuronal compartments from hBMECs.

hBMECs form a BBB that prevents paracellular permeability and that *in vivo* restricts access of blood constituents to neuronal compartments (17, 18). We evaluated changes in the barrier function of hBMECs following ZIKV infection by assessing the transendothelial electrical resistance (TEER) (58) and fluorescein isothiocyanate (FITC)-dextran permeability (59) of hBMEC monolayers grown on Transwell inserts. We found no significant change in TEER of ZIKV-infected versus mock-infected hBMECs at 1 to 3 dpi (Fig. 6A). After establishing that Transwell monolayers were intact, we disrupted paracellular hBMEC junctions with EDTA and found an ~100-Ω decrease in the TEER of hBMEC monolayers. Consistent with the TEER findings, the permeability of hBMECs to FITC-dextran was not enhanced by ZIKV infection of hBMECs compared to responses of mock-infected hBMEC controls (Fig. 6B). Collectively, these findings indicate that the barrier integrity and permeability of hBMECs is not significantly altered by ZIKV infection.

### ZIKV apical and basolateral infection and release from hBMECs.

hBMECs form polarized monolayers with apical and basolateral surfaces that mimic lumenal and ablumenal capillary surfaces (58, 60). In order to assess ZIKV entry and release from polarized hBMECs, we grew hBMECs for 5 days on Transwell inserts, infected hBMECs with ZIKV from the apical or basolateral sides, and assessed ZIKV titers in the upper and lower chambers at 1 dpi. ZIKV infection of either the apical or basolateral surfaces resulted in ZIKV release from both apical and basolateral hBMEC surfaces (Fig. 6C), while inoculation controls and TEER revealed no leakage across hBMEC monolayers. These results suggest that ZIKV infects and is released from both the lumenal and ablumenal sides of hBMECs. This observation provides a potential mechanism for ZIKV to cross hBMEC barriers and spread to neuronal compartments (Fig. 6D).

## DISCUSSION

ZIKV is distinguished from other mosquito-borne FVs by its unique ability to be sexually transmitted, cross placental and blood-brain barriers, cause *in utero* microencephaly, and persist for up to 6 months in patients (6, 12, 15, 16, 61). During human infections, ZIKV is found in a variety of body fluids (tears, saliva, semen, cervical mucus, and urine) that facilitate viral replication and dissemination, and ZIKV damages placental, corneal, and neuronal tissues (6, 12, 62). However, beyond the acute phase of infection, its persistence suggests that ZIKV uniquely replicates in cellular reservoirs where it balances cell survival and viral replication and evades innate and adaptive immune responses for extended periods. Persistence alone is likely to facilitate the ability of ZIKV to be sexually transmitted, spread across the placenta, and gain access to fetal and neuronal tissues. Our findings indicate that ZIKV infects primary hBMECs without the cytopathology that is reported for ZIKV-infected neurons and placental tissues (6). Cell death, observed following ZIKV infection of Vero E6 cells, was noticeably absent in ZIKV-infected hBMECs even at 9 dpi and after serial passage of infected hBMECs. This suggests the potential for persistently infected hBMECs to serve as cellular reservoirs for ZIKV replication and enable viral spread across BBBs into neuronal compartments.

Permeabilizing the endothelium is one mechanism for viruses to bypass EC barriers (21, 45). We found that ZIKV induced Rnd1 and ARHGAP26Rho in infected hBMECs, which direct brain capillary permeability (56, 57). These findings suggested potential mechanisms for ZIKV to permeabilize and spread across the BBB. However, when we evaluated hBMEC permeability, we found no significant difference between control and ZIKV-infected hBMEC monolayers. Instead, we found that ZIKV exited from apical and basolateral surfaces of hBMECs, suggesting a discrete mechanism for ZIKV to enter neuronal compartments via basolateral release and for systemic spread of ZIKV that is released apically from hBMEC reservoirs. Infection of HUVECs and other EC sites also provides prospective mechanisms for ZIKV to cross testicular, placental, and retinal EC barriers in ZIKV patients.

Successful viral pathogens have evolved mechanisms to evade antiviral innate immune responses. We found that ZIKV infection of primary hBMECs was inhibited by prior IFN-α addition, but that ZIKV-infected hBMECs were highly resistant to IFN-α by 3 hpi. In addition, ZIKV continued to spread within hBMECs for 1 to 9 dpi without apparent paracrine IFN restriction. In contrast, DENV spread in HUVECs was blocked by IFN-β secreted from DENV-infected cells (1 to 3 dpi) and relieved by an IFN-β antibody blockade (63). ZIKV’s resistance to IFN and its spread in hBMECs suggests that ZIKV employs discrete mechanisms to regulate innate hBMEC responses.

ZIKV-directed changes in hBMECs were initially addressed by kinetically analyzing global transcriptional responses to synchronous ZIKV infections. IFN-β and IFN-λ were transiently induced 1 to 2 dpi but, consistent with ZIKV persistence and spread in hBMECs, we failed to detect secreted IFNs in cell supernatants (Fig. 5). The absence of IFN does not appear to be due to altered secretion, as the CCL5/RANTES chemokine was both induced and highly secreted by ZIKV-infected hBMECs, and hBMECs were fully competent in secreting high levels of IFN-β in response to double-stranded RNA (Fig. 5B). In contrast, a subset of antiviral ISGs were highly induced and constitutively expressed in infected hBMECs 1 to 9 dpi (Table 1). In the absence of type I IFN secretion, ISG induction might be explained by the activation of existing IRFs or by ZIKV inducing IRF7 and IRF9 1 to 9 dpi (Table 1) (64), as IRF9 reportedly induces ISGs, including ISG15, without IFN and IFNAR-directed STAT1/2 phosphorylation (50).

ZIKV regulation of IFN induction and INFAR signaling pathways has been reported, and both ZIKV and DENV NS5 proteins regulate IFNAR-directed STAT2 signaling responses (20, 30, 65). STAT2 regulation may contribute to ZIKV resistance to added IFN-α, but this fails to explain ZIKV spread in partially infected monolayers, unless IFN expression is also inhibited. Our findings are supported by a recent study suggesting that ZIKV (PRVABC59) translationally restricts type I IFN in primary human dendritic cells (DCs) (28). However, findings that only ~8% of DCs were infected and that an INFAR antibody blockade increased ZIKV titers by 300% suggest that IFN is expressed and that ZIKV spread is severely restricted in DCs. Potential mechanisms of posttranscriptional IFN regulation during ZIKV infection remain to be defined and factored into an understanding of ZIKV persistence in hBMECs.

*In vitro*, ZIKV productively infects and induces ISGs in DCs, skin cells, macrophages, and trophoblasts (11–13, 28, 39, 66). Comparison of transcriptional results across studies is complicated by the different ZIKV strains used and in some studies analysis of cells showed that only 2 to 10% ZIKV infected, potentially amplifying paracrine IFN-directed ISG responses of bystander cells. In synchronously infected hBMECs (>80% infected), a large subset of antiviral ISGs were induced 1 to 9 dpi, that are seemingly at odds with ZIKV persistence in hBMECs. IFIT1, which interferes with *Alphavirus* translation (67), was highly induced and expressed in ZIKV-infected hBMECs without apparent consequence to ZIKV replication or persistence. In contrast, IFITM1, which is suggested to inhibit early ZIKV infection, was induced at very low levels by ZIKV while IFITM3, which is reported to prevent ZIKV-induced HeLa cell death (68), was not induced in ZIKV-infected hBMECs. These findings suggest that ZIKV employs novel mechanisms that selectively restrict antiviral ISG responses in order to persistently replicate and spread in hBMECs.

The lack of cytopathology during persistent ZIKV infection of hBMECs indicates that cell survival responses are engaged and that apoptotic responses are restricted in ZIKV-infected hBMECs. Several prosurvival genes are induced during ZIKV infection of hBMECs, including EGR1, ATF3, and BIRC3 (52, 53), and the constitutively induced chemokine CCL5 (40- to 2,327-fold 1 to 9 dpi) is also associated with cell survival (69–72). In contrast, IRF1, which directs apoptosis when expressed constitutively, was only transiently induced, while XAF1, a protein that inactivates inhibitor of apoptosis proteins (IAPs; BIRC2/3), was continuously induced along with its regulatory target, IAP2 (55, 73). Additional prosurvival responses may also be directed by ZIKV-engaging Axl receptors, as Axl performs proliferative and cell survival functions and is linked to IFNAR regulation (48, 74). These findings suggest that ZIKV establishes persistence by balancing induced apoptotic and cell survival responses in hBMECs.

HCV replicates at low levels in persistently infected hepatocytes by inducing an IFN-resistant state and promoting cell survival (24, 25). With HCV, ISG15 induction is actually proviral and contributes to HCV persistence and IFN resistance in hepatocytes (26, 27, 51). ISG15 knockdown renders HCV-infected cells susceptible to IFN regulation, and human cells devoid of ISG15 have enhanced antiviral protection that is lost by expressing ISG15 (26, 75). Further proviral ISG15 functions stem from human ISG15 interactions, which are crucial for USP18-mediated inhibition of IFN-α/β signaling responses (49). Consistent with prosurvival roles for ISG15, ZIKV-infected hBMECs constitutively induce ISG15, ISG15 ligases (HERC5/6), and ISG15 proteases (USP11/18/41). ZIKV induction of the ISG15 family further suggests the potential for USP18-directed downregulation of IFN-α/β translation and for HERC5 to enhance IRF stability that may drive ISG induction during ZIKV infection (49, 75, 76). Whether ZIKV cytopathology is linked to the absence of ISG15 in Vero cells (77) remains to be determined, as do proviral or IFN regulatory roles for ISG15 in ZIKV-infected hBMECs.

CCL5 is a chemokine linked to antiapoptotic responses (70, 72, 78), and CCL5 was the only chemokine highly induced and constitutively secreted by ZIKV-infected hBMECs (1 to 9 dpi). CCL5 responses are directed by WNV, DENV, and Tick-borne encephalitis virus (TBEV) infection of brain ECs, and CCL5 induction is associated with increased WNV and TBEV pathogenesis (79, 80). On the EC surface, CCL5 forms a filamentous complex that is key to the recruitment of immune cells and activated CD4^+^ T cells to human BMECs (70, 72). Although the half-life of CCL5 on the surface of ECs is only 30 min, constitutive CCL5 secretion during persistent ZIKV infection of hBMECs may traffic immune cells to the BBB, and both foster ZIKV’s spread to neurons and inflammatory CNS pathology (69, 71, 78). Further studies are required to determine whether transcytosis is an additional means of ZIKV crossing hBMEC barriers (81).

RNA viruses which lack DNA intermediates are rarely associated with extended or persistent infections. Measles and rubella viruses are examples of RNA viruses that persist and cause neuronal disease (60, 82, 83), but mechanisms of persistence remain perplexing. While ZIKV infection does not establish HCV-like permanence, ZIKV persistence in hBMECs without cytotoxicity suggests that hBMECs are potential ZIKV reservoirs that foster viral spread and pathogenesis. Nevertheless, ZIKV may also persist in additional cell types and compartments. Recent studies suggested that human placental macrophages (Hofbauer cells) and DCs are 90% viable after ZIKV (PRVABC59) infection (13, 28). However, it remains to be determined whether ZIKV directs survival responses or persistently infects these cells, as only 5% of Hofbauer cells and 2 to 8% of DCs were infected in these studies. In addition to discovering other persistently infected cell types, studies of ZIKV evasion of adaptive immune responses are required in order to understand ZIKV persistence mechanisms *in vivo* (84). A recent *in vivo* study correlated persistent ZIKV infection of rhesus monkey CNS and lymph nodes with transcriptomic responses of peripheral blood mononuclear cells (40). This study noted the induction of ATF3, EGR1, JUNB, IRF7, ISG15, HERC5/6, additional ISGs, chemokines CCL5 and CXCL10, prosurvival responses, and decreased proapoptotic genes, similar to gene induction levels we found to be induced by ZIKV infection of hBMECs (Table 1) (GEO GSE98889).

Strategic roles for ECs in ZIKV persistence and spread are suggested by the wide range of restricted tissues targeted by ZIKV. ECs control immune cell recruitment, targeting, and transcytosis into tissues and perform primary fluid barrier functions that prevent capillary leakage. ECs innervate and protect immune-restricted compartments from viral spread (17), and emigration across ECs is required for ZIKV to bypass blood-retina barriers and blood-testicle barriers to infect retinal ganglion cells, aqueous humor, the cornea, Sertoli cells, seminiferous tubules, and spermatagonia (18, 43, 44). ECs express tolerizing PD-L1 receptors that prevent immune targeting of the endothelium and which may foster viral immune evasion and persistence (85). Our findings define hBMECs as ZIKV reservoirs that permit lumenal emigration and spread within blood and potentially provide ZIKV the ability to cross brain EC barriers and enter neuronal compartments. Remarkably, ZIKV persists in hBMECs with little or no cytopathology through ZIKV-directed cell survival responses. These findings suggest mechanisms for ZIKV persistence as well as potential hBMEC targets for restricting ZIKV persistence and spread.

## MATERIALS AND METHODS

### Cells and virus.

Vero E6 cells (ATCC CRL 1586) were grown in DMEM (Dulbecco’s modified Eagle’s medium) supplemented with 8% fetal bovine serum (FBS) and penicillin (100 μg/ml), streptomycin sulfate (100 μg/ml), and amphotericin B (50 μg/ml; Mediatech) at 37°C and 5% CO_2_. hBMECs (passage 3), derived from elutriation of dispase-dissociated normal human brain cortex tissue were purchased from Cell Biologics (H-6023). HUVECs were purchased from Cambrex, and the immortalized hCMEC/D3 cell line was purchased from Cedarlane Labs. Inc. hBMECs (passages 4 to 10), HUVECs (passages 3 to 8), and hCMEC/D3 cells were grown in endothelial cell basal medium-2 MV (EBM-2 MV; Lonza) supplemented with EGM-2 MV SingleQuots (Lonza) and incubation at 37°C and 5% CO_2_.

ZIKV (PRVABC59) was obtained from the ATCC, minimally passaged (MOI, 0.1 to 1), and propagated for 5 days in Vero E6 cells in DMEM with 2% FBS. ZIKV was allowed to adsorb to ~60% confluent hBMEC monolayers for 2 h. Following adsorption, monolayers were washed with phosphate-buffered saline (PBS) and grown in supplemented EBM-2 MV with 5% FBS. Vero E6, HUVEC, and hCMEC/D3 cells were similarly infected, washed, and supplemented with DMEM with 8% FBS. ZIKV titers were determined by serial dilution and infection of Vero E6 cells, quantifying infected cell foci at 24 hpi by immunoperoxidase staining with anti-DENV4 hyperimmune mouse ascites fluid (HMAF; 1:1,200; ATCC), horseradish peroxidase (HRP)-labeled anti-mouse IgG (1:2,000; KPL-074-1806), and 3-amino-9-ethylcarbazole staining (59, 86). For IFN-α inhibition studies, medium was supplemented with 1,000 U/ml IFN-α (Sigma-Aldrich) at indicated times and cells incubated at 37°C and 5% CO_2_. For analysis of apical and basolateral release of ZIKV, hBMECs were grown for 5 days on Costar Transwell inserts (3 μm) and evaluated for confluence by T (EVOM2, STX3; World Precision Instruments, Inc.). hBMECs were ZIKV infected apically or basolaterally (MOI, 5), washed with PBS and the medium, and incubated for 24 h prior to analyzing viral titers in apical and basolateral supernatants.

### CyQuant cell viability assay.

ZIKV (MOI, 10) or mock-infected hBMECs or Vero E6 cells were assayed for viability using a CyQuant NF cell proliferation assay kit (Thermo-Fisher Scientific). Medium was removed from mock- or ZIKV-infected monolayers (MOI, 10) at the indicated day postinfection and replaced with CyQuant reaction mixture. Wells were incubated for 15 min before fluorescence was quantified using a BioTek FLx800 fluorimeter (490-nm excitation, 530-nm emission). Fluorescence units of ZIKV-infected versus mock-infected cells were compared to determine the percent fluorescence representing relative cell viability.

### Live/dead assay.

PI (Calbiochem) and calcein-AM (Invitrogen) uptake were used to evaluate hBMEC and Vero E6 cell viability. ZIKV-infected (MOI, 10) or mock-infected hBMECs were seeded into 96-well plates and at the indicated times costained by the membrane-permeable dye calcein-AM (3 μM; green fluorescence in live cells), and 2.5 μM propidium iodide (red fluorescent DNA stain to detect dead cells). Images of calcein-AM-positive versus PI-positive cells were resolved using an Olympus IX51 microscope and Olympus DP71 camera and overlayed using Adobe Photoshop.

### Affymetrix gene array analysis.

Primary hBMECs (passages 5 to 10; Cell Systems) were synchronously infected with ZIKV (PRVABC59) (MOI, 20) or mock infected. Mock- or ZIKV-infected hBMECs were lysed, and total RNA was purified 12 hpi to 9 dpi by using RNeasy (Qiagen). Purified RNA was quantitated, and transcriptional responses were detected with Affymetrix Clariom-S chip arrays in the Stony Brook Genomics Core Facility. ZIKV-infected cell transcriptional responses were compared to those in mock-infected cells harvested at each time pointm and fold changes in ZIKV versus control hBMEC transcripts were analyzed by using the Affymetrix TAC software. Data obtained from these studies were submitted to the NCBI Gene Expression Omnibus database (GEO GSE98889).

### qRT-PCR analysis.

Quantitative real-time PCR was performed on purified RNAs from mock- or ZIKV-infected hBMECs as described above. cDNA synthesis was performed using a Transcriptor first-strand cDNA synthesis kit (Roche) using random hexamers as primers (25°C for 10 min, 50°C for 60 min, and 90°C for 5 min). qRT-PCR primers for specific genes were designed according to the NCBI gene database with 60°C annealing profiles (provided by Operon). qRT-PCR primers to ZIKV (PRVABC59) RNA were as follows: forward, CCGTGCCCAACACAAG; reverse, CCACTAACGTTCTTTTGCAGACAT. Genes were analyzed using PerfeCTa SYBR green SuperMix with ROX (Quanta Biosciences) on an ABI 7300 real-time PCR system (Applied Biosystems). Responses were normalized to internal glyceraldehyde-3-phosphate dehydrogenase (GAPDH) mRNA levels, and the fold induction was calculated using the 2^−ΔΔ*CT*^ method for the difference between mock- and ZIKV-infected hBMEC RNA levels at each time point.

### Transendothelial electrical resistance.

hBMECs were plated on Costar Transwell inserts (3-μm pore size; Corning) at high density, and 5 days postseeding hBMEC monolayers were analyzed for TEER (EVOM2; STX3; World Precision Instruments, Inc.). Confluent Transwell cultures were infected with ZIKV (MOI, 5) or mock infected, and TEER values for ZIKV-infected versus mock-infected hBMECs were compared at 1 to 3 dpi. To assess Transwell monolayer resistance, 10 mM EDTA was added to apical supernatants for 10 min, and TEER was assessed before and after this addition. EDTA treatment reduced TEER by ~100 Ω, demonstrating the resistance change directed by disrupting interendothelial cell junctions.

### FITC-dextran permeability analysis.

A gold standard Transwell permeability assay was used to assess the permeability of ZIKV-infected hBMECs (59) on Costar Transwell plates in triplicate. Transwells were seeded and infected (MOI, 5) or mock infected as described above. At 3 dpi, FITC-dextran (40 kDa; 0.5 mg/ml; Sigma) was added to the upper chamber and the levels of FITC-dextran observed in the lower chamber were monitored over time by using a BioTek FLx800 fluorimeter (490-nm excitation, 530-nm emission). FITC-dextran fluorescence directed by transit across ZIKV-infected or mock-infected hBMECs was quantitated (59). The data presented represent results of three independent experiments.

### ELISA.

Levels of IFN-β, IFN-λ, and CCL5/RANTES in the supernatants of mock- and ZIKV-infected hBMECs at 12 hpi to 9 dpi were measured using a DuoSet ELISA (R&D Systems). hBMECs transfected with poly(I/C) (1 µg/ml) and Fugene6 (3:1; Promega) were similarly evaluated for secreted IFN-β at 36 h posttransfection. ELISA plates (Immunolon 2, U-bottom; Dynatech Laboratories) were coated with anti-IFN-β, anti-IFN-λ, or anti-CCL5 ELISA reagents according to the manufacturer. Viral supernatants were incubated on coated plates (2 h) and washed with PBS (0.1% Tween 20), and bound protein was detected with target-specific antibodies conjugated to streptavidin-HRP and developed using tetramethylbenzidine. Protein concentrations were determined based on the optical density, using a BioTek EL312e microplate reader (450 nm), and compared to standard curves for purified IFN-β, IFN-λ, and CCL5 (R&D Systems).

### Western blotting.

Western blot assays were performed as previously described (86). Briefly, MECs were infected with ZIKV (MOI, 10) or mock infected and harvested at 1 to 9 dpi. Cells were washed with PBS followed by lysis in buffer containing 1% NP-40 (150 mM NaCl, 50 mM Tris-Cl, 10% glycerol, 2 mM EDTA, 10 nM sodium fluoride, 2.5 mM sodium pyrophosphate, 2 mM sodium orthovanadate, 10 mM β-glycerophosphate) with protease inhibitor cocktail (Sigma). Total protein levels were determined in a bicinchoninic acid assay (Thermo Scientific), and 20 µg of protein was resolved by SDS–12% polyacrylamide gel electrophoresis. Proteins were transferred to nitrocellulose, blocked in 5% bovine serum albumin, and incubated with the indicated antibodies. Antibodies used were anti-ZIKV envelope (GTX133314; GeneTex), anti-IFIT1 (Santa Cruz Biotechnology), anti-MxA (D3W71; Cell Signaling), and anti-GAPDH (G9545; Sigma-Aldrich). Protein was detected using HRP-conjugated anti-mouse or anti-rabbit secondary antibodies (Amersham) and Luminata Forte Western HRP substrate (Millipore).

### Statistical analysis.

Results shown in each figure were derived from two to three independent experiments with comparable findings; the data presented are means ± standard errors of the means (SEM), with the indicated *P* values of <0.01 and <0.001 considered significant. Two-way comparisons were performed two-tailed analysis of variance and an unpaired Student’s *t* test. All analyses were performed using GraphPad Prism software version 4.0.
