# Supplementary figures and images for: Zika Virus Persistently Infects and Is Basolaterally Released from Primary Human Brain Microvascular Endothelial Cells
Source: mBio. 2017 Jul 11;8(4):e00952-17. doi: 10.1128/mBio.00952-17 (PMC5513708; doi:10.1128/mBio.00952-17)

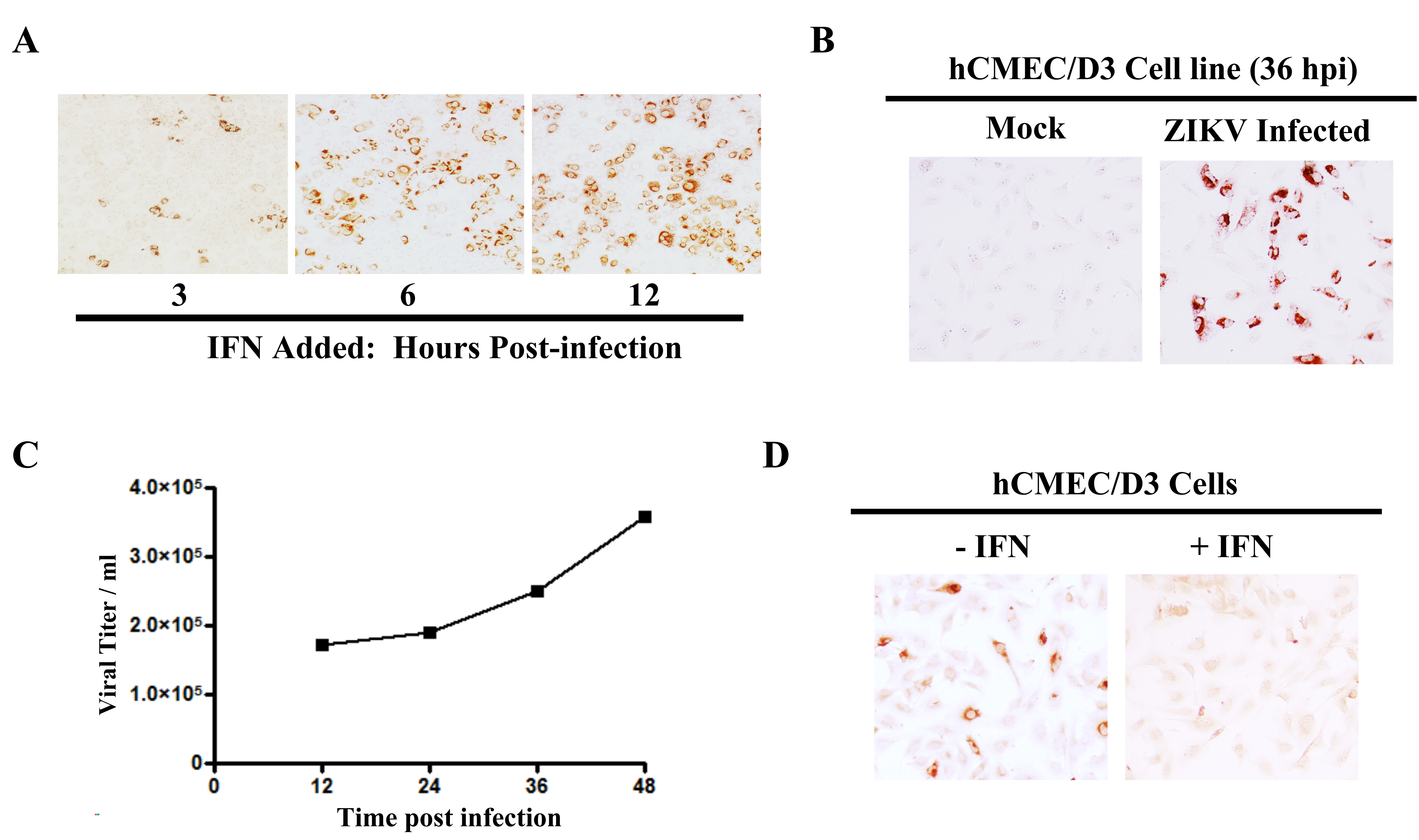

Supplement: FIG S1 [file mbo003173371sf1.tif]

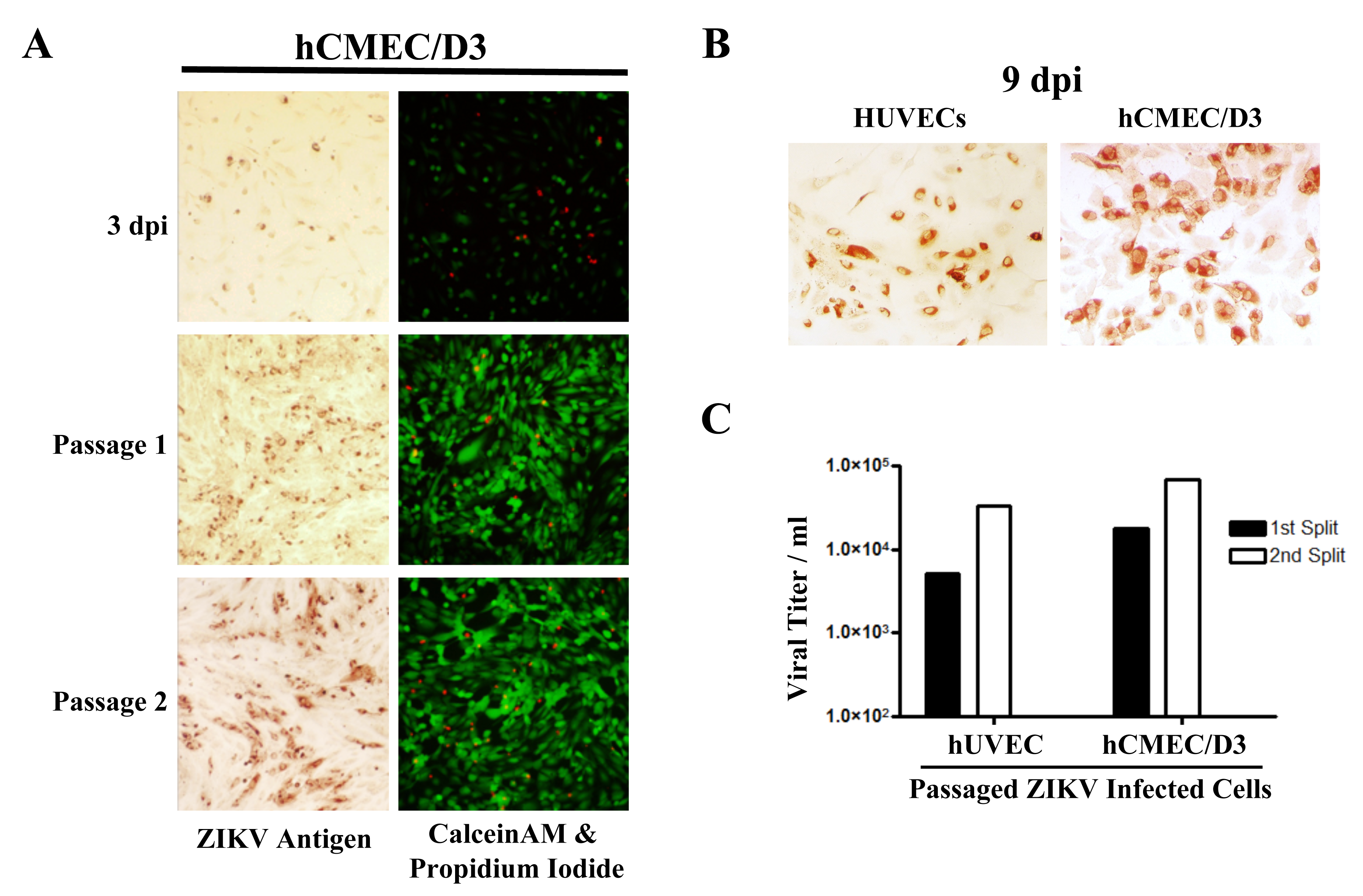

Supplement: FIG S2 [file mbo003173371sf2.tif]

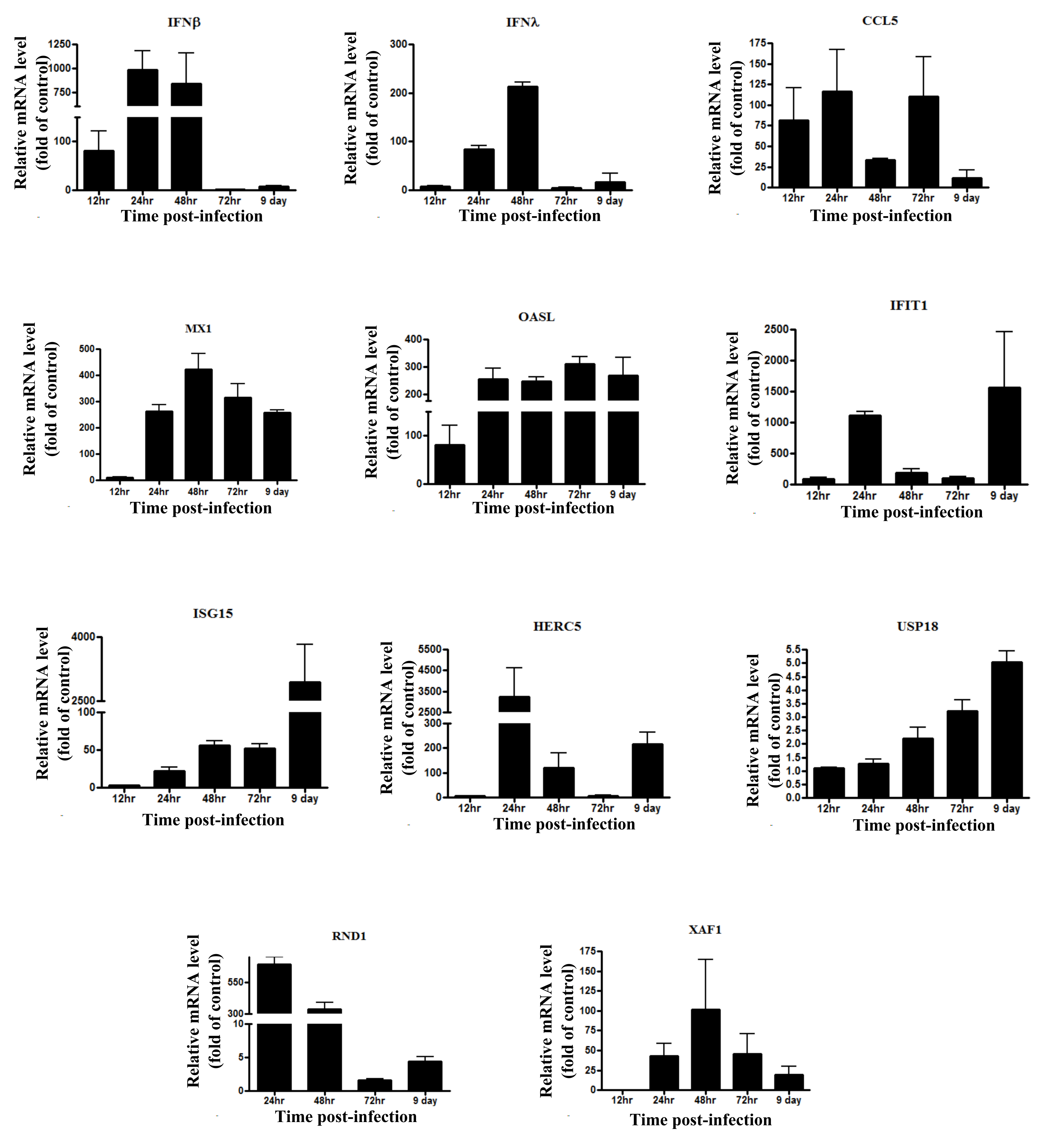

Supplement: FIG S3 [file mbo003173371sf3.tif]
